# Cryptogenic Stroke and Significance of the Patent Foramen Ovale: A Case Series

**DOI:** 10.7759/cureus.3525

**Published:** 2018-10-30

**Authors:** Issa Pour-Ghaz, Rashi Krishnan, William F Pierce, Christopher D Jackson, Rohini Bhole, Ankur Seth

**Affiliations:** 1 Internal Medicine, University of Tennessee Health Science Center, Memphis, USA; 2 Neurology, University of Tennessee Health Science Center, Memphis, USA; 3 Neurology, Methodist University Hospital, Memphis, USA

**Keywords:** patent foramen ovale, cryptogenic stroke, transient ischemic attack, pfo, pfo closure

## Abstract

Stroke is the second leading cause of death globally and can lead to significant adverse outcomes in patients following the acute illness. Due to this high morbidity and mortality, adequate interventions can play a significant role in health outcomes. Patent foramen ovale is one of the major proposed causes of cryptogenic strokes and can be present in up to 25% of general population. In cryptogenic strokes, the relation of this structural heart defect is inversely proportional to age of patient. Here, we present three cases of cryptogenic strokes in patients with patent foramen ovale where it possibly plays a significant role. We demonstrate that in the younger age spectrum, patent foramen ovale plays a more significant role and treatment could prevent future stroke episodes.

## Introduction

Stroke is the second leading cause of death globally and one of the important causes of morbidity and mortality in the United States [1]. Cryptogenic strokes consist of a subset of strokes where no probable cause is identified despite adequate diagnostic evaluations. Cryptogenic stroke is a diagnosis of exclusion and can only be diagnosed after workup for other causes such as large artery atherosclerosis, small artery disease, cardioembolic and structural cardiac abnormalities, has been ruled out [2]. Cryptogenic strokes can be subclassified into ‘highly cryptogenic’ and those of ‘possibly determined origin’ [3]. Cryptogenic strokes account for approximately 25% of strokes, which is a significant number of cases without an identifiable cause, thus, treating patients for secondary prevention is essential. Hence, the term cryptogenic stroke is used to describe the absence of any other known cause of stroke except patent foramen ovale (PFO). Moreover, it has been established that PFO prevalence is significantly higher in patients who have cryptogenic strokes [4]. As a result, when a cryptogenic stroke is an underlying cause for stroke, closure of the PFO would be the next step. However, there have been multiple randomized trials in the earlier years which failed to show the benefit of PFO closure. More recently, trials have shown that long-term outcome of PFO closure reduces the recurrence of strokes. CLOSE trial (Patent Foramen Ovale Closure or Anticoagulants versus Antiplatelet Therapy to Prevent Stroke Recurrence; NCT00562289) showed that percutaneous PFO closure was associated with significantly fewer recurrent strokes in patients with cryptogenic stroke which was also the end result of the RESPECT trial (Randomized Evaluation of Recurrent Stroke Comparing PFO Closure to Established Current Standard of Care Treatment; NCT00465270). RESPECT trial showed that PFO closure significantly reduced the incidence of recurrent stroke compared with medical therapy [5, 6]. It is important to note that choosing which patients get PFO closure through risk stratification is vital for such patients after adequate workup is done to exclude other causes for stroke. Here, we will present three cases that demonstrate why early PFO closure, especially in younger patients, can be beneficial.

## Case presentation

Case 1

**Figure 1:**
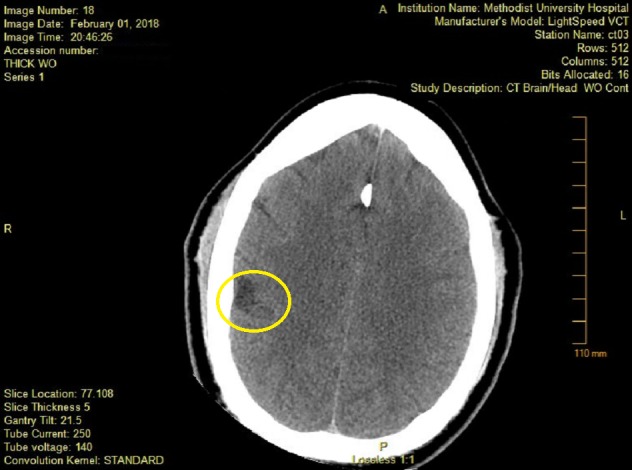
Computed tomography (CT) head on admission showing subacute right parietal cortical hypodensity. Area of interest marked by circle.

A 30-year-old African American male with past medical history of insulin dependent diabetes mellitus, hypertension, sleep apnea and a recent myocardial infarction seven months prior presents with acute onset dysarthria and angioedema secondary to lisinopril use. His angioedema was treated appropriately, and stroke team was consulted for concerns due to dysarthria. The patient also commented that he was having intermittent right-sided upper extremity paresthesias. A non-contrast computer tomography (CT) of head was obtained, which demonstrated a subacute right parietal cortical hypodensity (Figure 1). Since his previous myocardial infarction, the patient had complaint with his aspirin and Plavix. He noted that approximately one month ago, he suffered a severe headache, but did not remember which side it was on, what precipitated this headache or how long it lasted. Review of systems was negative except for the angioedema and dysarthria. His labs on presentation were low-density lipoprotein (LDL) 123, HbA1c 7.2, hemoglobin and hematocrit of 20.9 and 65.7 with P2Y12 88. A magnetic resonance imaging (MRI) was performed, which showed no evidence of diffusion restriction to suggest recent infarction. Encephalomalacia was demonstrated involving the right parietal lobe secondary to sequela from remote infarction. Computer tomography angiography (CTA) was negative for any pathology including any significant stenosis. Transthoracic echocardiogram (TTE) showed a left ventricle (LV) with normal size and normal systolic function. LV ejection fraction was 55-60% with normal regional wall motion, normal diastolic function, and no LV thrombus. There was no evidence of atrial septal defect, ventricular septal defect or PFO. At this time the recommendations were cardiac event monitor and outpatient follow-up since symptoms were consistent with a transient ischemic attack (TIA) versus dysarthria secondary to the angioedema, both of which required no intervention.

**Figure 2:**
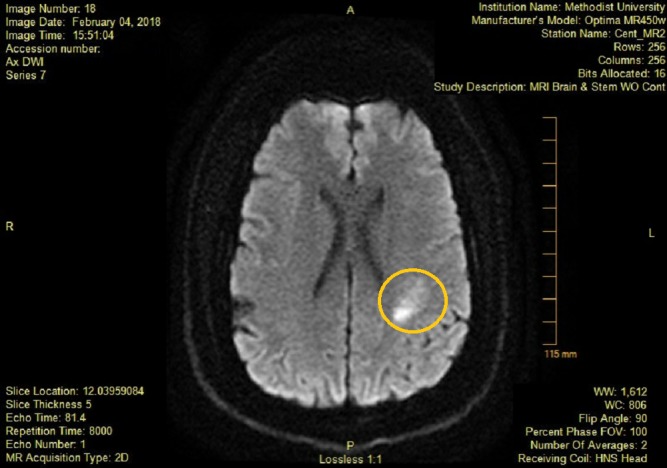
DW-MRI obtained after stroke symptoms showing new area of enhancement. Area of new stroke marked by circle. DW-MRI: Diffusion-weighted magnetic resonance imaging.

**Figure 3:**
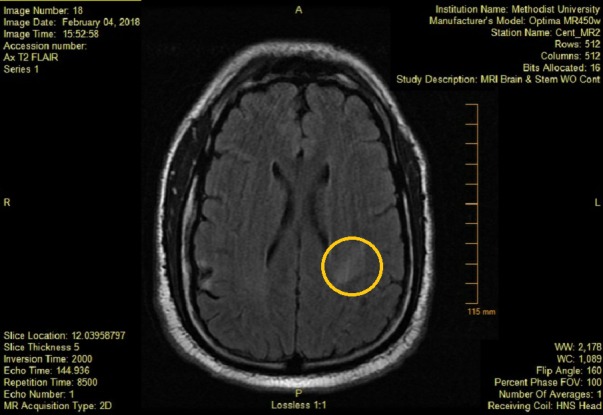
MRI FLAIR showing new area of enhancement after symptoms of stroke. Area of interest marked by circle. MRI FLAIR: Magnetic resonance imaging fluid-attenuated inversion recovery.

Approximate 36 hours after stroke team visit, the patient was found to have a right-sided facial droop and right-sided arm weakness with last known well reportedly overnight. NIHSS was 4 (right facial droop, dysarthria, right upper extremity drift) and stroke team was re-consulted. MRI showed interval development of diffusion restriction involving the left parietal lobe and there was associated signal hypo-intensity on the apparent diffusion coefficient map most characteristic of recent infarction (Figures 2, 3). This acute left parietal small ischemic stroke was out of window for tissue plasminogen activator administration. Transesophageal echocardiogram (TEE) with saline contrast injection was performed at rest with good effort Valsalva which demonstrated at least 20 early appearing left heart bubbles, consistent with present of a large size PFO. Magnetic resonance angiography showed no evidence for pelvic or common femoral venous thrombosis. The stroke was attributed to a large inter-atrial communication defect with etiology being paradoxical embolus through defect. The patient was considered for transcatheter PFO closure, however, he opted for outpatient closure of defect. He was sent home with close follow-up and event monitor together with atorvastatin and dual antiplatelet therapy.

Case 2

**Figure 4:**
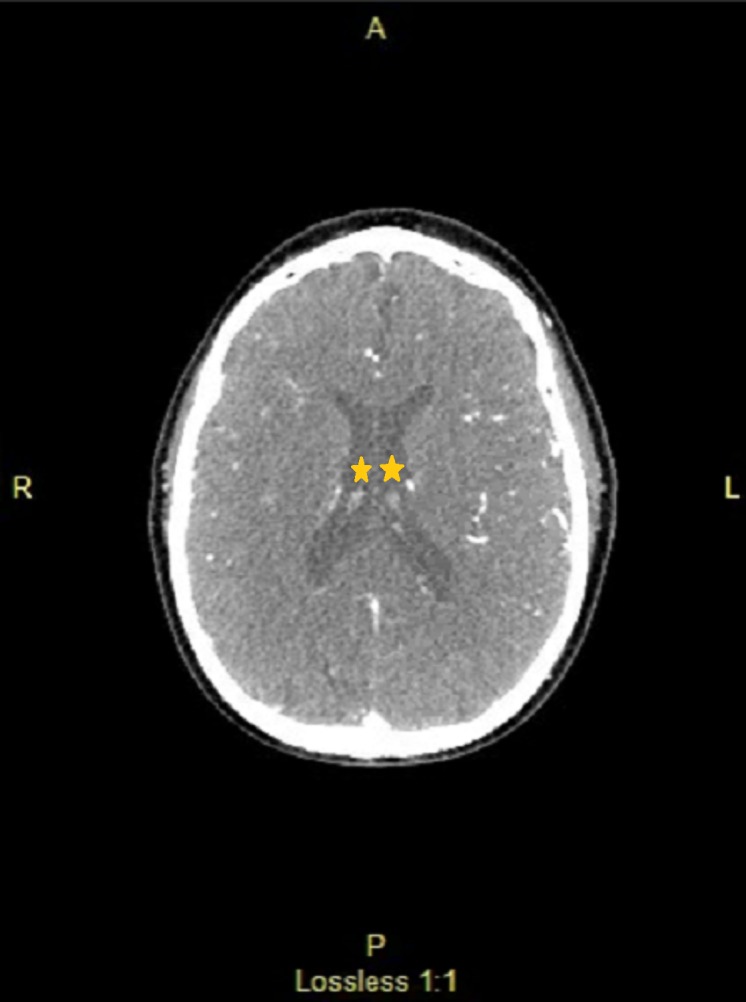
Computed tomography (CT) head showing no acute bleeding and no midline shift. Ventricles marked by star showing absence of bleeding or midline shift.

**Figure 5:**
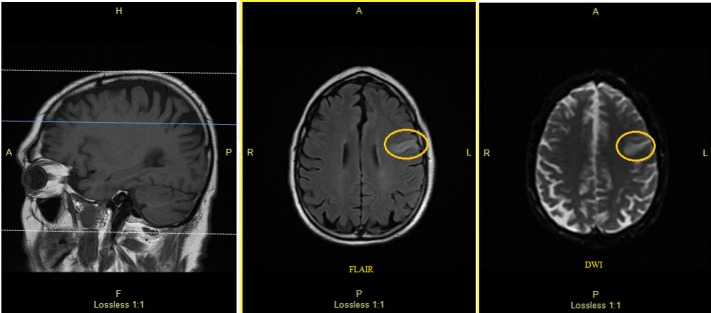
Magnetic resonance imaging (MRI) of head without contrast at presentation showing acute left middle cerebral artery territory infarction without midline shift or hemorrhagic conversion. Area of stroke marked by circles.

A 66-year-old Caucasian male with past medical history significant for hypertension and hyperlipidemia presented with altered mental status. Symptoms at presentation included slurred speech. He was found walking around his yard and his neighbor noted his slurred speech. His last know well state was the day before in the evening hours. Upon arrival, the blood pressure was found to be at 150/70 and his laboratory workup including complete blood count, complete metabolic profile and urinalysis were normal. Urine drug screen was positive for cannabinoids and serum alcohol level was not detectable. CT of the head showed age-related atrophy and mild small vessel ischemic changes without acute intracranial bleeding (Figure 4). Questionable loss of gray-white junction and edema was found in the left frontoparietal region. On physical exam, the patient was confused but had normal movements and strength. He denied use of any tobacco products. TTE showed a normal left ventricle size, thickness, and function. The ejection fraction was estimated at 60% with normal right ventricular structure and function. There was a small PFO with predominant left-to-right shunting visualized. MRI showed an acute left middle cerebral artery territory infarction without midline shift or hemorrhagic conversion (Figure 5). CTA showed the right internal carotid artery with estimated stenosis of 30-50% and the left internal carotid artery with stenosis estimated at 30-50%. TEE showed the PFO previously demonstrated and an ejection fraction of approximately 60%. Ultrasound of lower extremities failed to show deep vein thrombosis. The patient continued receiving neuro checks during the length of the stay and was started on antiplatelet therapy and atorvastatin with an outpatient follow-up and discharged with an event recorder and possible percutaneous closure of PFO after conduction abnormalities were ruled out.

Case 3

An 18-year-old Caucasian female with past medical history significant for Raynaud’s phenomena and vascular headaches, which have never been associated with any neurologic abnormalities, presented after an episode of not being able to articulate and not being able to feel her right arm and the right side of her face. During this episode, the patient had a unilateral frontal headache and a brief episode of lightheadedness. The episode lasted two minutes after which she returned to baseline. The patient was on oral contraceptive pills until this episode. On examination, the patient did not have any significant physical findings. TTE showed a normal ejection fraction of 65% with normal diastolic function and the presence of a PFO. CTA brain with and without contrast did not show any stenosis, dissection, or aneurysm. MRI of the brain with and without contrast showed no acute intracranial findings. The patient underwent the placement of an Amplatzer closure device and was followed up with repeat TEE which showed an ejection fraction greater than 55% and no evidence of spontaneous echo contrast in the left atrium. Repeat echocardiography showed that the Amplatzer device was in good position. On follow-up visits, the patient did not have any new episodes of a cryptogenic stroke. The patient had the last echocardiography done in February 2018, which showed that the ejection fraction was greater than 65% and that the atrial septal occluder device was in a stable position with no residual shunting. There was no evidence of device migration. The remaining valves were grossly normal. Since the placement of the closing device, the patient has been doing well without any residual neurologic defects or repeated episodes of TIA or stroke. RoPE score measured for this patient was 9 with an 88% chance of stroke being due to a PFO.

## Discussion

Ischemic strokes are divided into multiple categories including large artery atherosclerosis, small vessel disease, and cardioembolic strokes which make up majority of the identifiable causes. One of the most important factors for determining a stroke cause is the age of the patient. In younger population, specifically those less than 60-year-old, hematologic and structural causes are more common while arteriolosclerosis and atrial fibrillation are more common in individuals older than 60-year-old. Cryptogenic strokes are defined as any stroke that cannot be attributed to any specific cause despite adequate investigation. This can account for up to 30% of the strokes. Patients presenting to the hospital with symptoms of stroke must undergo investigation to determine the underlying cause due to the importance of time in stroke management. This involves performing an adequate history and physical examination and utilization of adequate imaging and laboratory modalities. Some of the standard evaluation tools used in stroke assessment include CT of the brain, MRI of the brain, vessel imagining using magnetic resonance angiography (MRA) or CTA, TTE, TEE, cardiac rhythms and complete blood count. If these modalities fail to establish the cause of stroke, then patients require further investigation such as a prolonged duration of cardiac rhythm monitoring and hematologic testing. When all these examinations fail to show a true cause for the stroke, then it is considered cryptogenic [7].

PFO is a connection between the left and right atria that can allow blood or blood clots to travel paradoxically from the right atria to the left. PFO is a common phenomenon and can be found in 25% of the general population [8]. Presence of PFO in patients who have cryptogenic strokes can range anywhere from 10 to 77% [9, 10]. The relationship becomes more striking when we look at patients under the age of 55 years who are more likely to have a PFO compared with patients who have a known cause of stroke [11]. After the first episode of cryptogenic stroke, risk of having a repeat stroke increases on average 2% annually. Studies have shown that this number can be as high as 16% [12]. It has already been established that in 50% of the cases, stroke in TIA patients occurs within 48 hours of the TIA and up to 15% have a stroke within three months, thus prompt assessment is crucial [13]. Hence, treatment and type of therapy plays an important role in these cases since patients who have an episode of stroke or TIA are much more likely to have a repeat episode of stroke which is even more true in those with PFO. In addition, Risk of Paradoxical Embolus (RoPE) calculator is a useful tool that can be used to check for the likelihood that the stroke and the PFO are related. It considers age (younger age gives more points), infarct on imaging, smoking history, stroke or transient ischemic attack history, diabetes history, and hypertension with a maximum score of 10. Due to the unique scoring feature, RoPE calculator can identify younger patients without conventional risk factors. The probability of finding a PFO increases from 23% (with scores 0 to 3) to 73% (with a score of 9 or 10), which correspond, respectively, to a PFO-attributable risk of 0% to 88%. This makes RoPE calculator a useful tool when presented with a case of cryptogenic stroke.

For case 1, in addition to poorly controlled comorbidities, polycythemia plays a significant role in thrombus formation causing strokes. Hence, the patient would benefit from PFO closure in addition to optimized medical treatment of comorbidities for stroke prevention. In case 2, the patient needs to be investigated for other etiologies of stroke such as arrhythmias with cardiac event recorders. Case 3 is a young patient with a history of migraine and oral contraceptive use, both being risk factors for developing TIA. However, majority of younger patients with TIA without significant comorbidities are at an increased risk of subsequent strokes and would benefit from PFO closure, as in this case.

Currently, majority of patients who have cryptogenic stroke and PFO are managed by antiplatelet and hyperlipidemia therapy. Until recently, in this category of patients, the consensus was not to close the PFO due to earlier studies not showing any benefit including CLOSURE I (Evaluation of the STARFlex Septal Closure System in Patients with a Stroke and/or Transient Ischemic Attack due to Presumed Paradoxical Embolism), PC (Percutaneous Closure of Patent Foramen Ovale in Cryptogenic Embolism), and RESPECT (Randomized Evaluation of Recurrent Stroke Comparing PFO Closure to Established Current Standard of Care Treatment). These trials failed to show superiority of closure over medical therapy [14-16]. Also, in a 2014 meta-analysis of randomized trials by Udell et al., it was concluded that at that time transcatheter closure of the PFO did not significantly reduce the short-term incidence of recurrent stroke or TIA [17]. However, more recently, there has been long-term follow-up of patients who have undergone PFO closure and there have been multiple new studies that have investigated PFO closure outcomes. Mas et al. and Søndergaard et al. demonstrated that PFO closure reduced risk for ischemic stroke and new brain infarctions without significant increase in serious adverse events [18, 19]. De Rosa et al. performed a systematic review and meta-analysis of PFO closure versus medical therapy in stroke patients with PFO and they concluded that compared to medical treatment, closure of PFO does prevent recurrent stroke and TIA [20].

## Conclusions

When considering PFO closure in stroke patients, especially those younger than 60 years old, it is important to rule out large artery atherosclerosis, cardioembolic source, small vessel disease, dissection and hypercoagulable states as the cause. Only when the stroke has been categorized as cryptogenic in patients with PFO, especially in younger patients, PFO closure should be strongly considered. Some of the factors that support PFO closure include previous thromboembolism, multifocal cerebral defects, a large PFO, or atrial septal aneurysm. For future directions of the PFO closure and studies, it would be beneficial to see what the subset analysis shows based on different age groups of patients who have undergone PFO closure and study its long-term morbidity and mortality benefit.
